# Biomedical Relation Extraction Using Dependency Graph and Decoder-Enhanced Transformer Model

**DOI:** 10.3390/bioengineering10050586

**Published:** 2023-05-12

**Authors:** Seonho Kim, Juntae Yoon, Ohyoung Kwon

**Affiliations:** 1 Department of Computer Science and Engineering, Sogang University, Seoul 04107, Republic of Korea; shkim.lex@gmail.com; 2 VAIV Company, Seoul 04107, Republic of Korea; 3 Department of Future Technology, Korea University of Technology and Education, Cheonan-si 31253, Republic of Korea

**Keywords:** DDI (drug–drug interaction), CPR (chemical–protein relation), transformer, self-attention, GAT (graph-attention network), relation extraction, ChemProt, T5 (text-to-text transfer transformer)

## Abstract

The identification of drug–drug and chemical–protein interactions is essential for understanding unpredictable changes in the pharmacological effects of drugs and mechanisms of diseases and developing therapeutic drugs. In this study, we extract drug-related interactions from the DDI (Drug–Drug Interaction) Extraction-2013 Shared Task dataset and the BioCreative ChemProt (Chemical–Protein) dataset using various transfer transformers. We propose BERT_GAT_ that uses a graph attention network (GAT) to take into account the local structure of sentences and embedding features of nodes under the self-attention scheme and investigate whether incorporating syntactic structure can help relation extraction. In addition, we suggest T5_slim_dec_, which adapts the autoregressive generation task of the T5 (text-to-text transfer transformer) to the relation classification problem by removing the self-attention layer in the decoder block. Furthermore, we evaluated the potential of biomedical relation extraction of GPT-3 (Generative Pre-trained Transformer) using GPT-3 variant models. As a result, T5_slim_dec_, which is a model with a tailored decoder designed for classification problems within the T5 architecture, demonstrated very promising performances for both tasks. We achieved an accuracy of 91.15% in the DDI dataset and an accuracy of 94.29% for the CPR (Chemical–Protein Relation) class group in ChemProt dataset. However, BERT_GAT_ did not show a significant performance improvement in the aspect of relation extraction. We demonstrated that transformer-based approaches focused only on relationships between words are implicitly eligible to understand language well without additional knowledge such as structural information.

## 1. Introduction

With the rapid progress in biomedical studies, it is a very challenging issue to extract efficiently useful information described in the biomedical literature. According to LitCOVID [1], over 1000 articles were published in just three months from December 2019, when COVID-19 was first reported, to March 2020. In PubMed [2] which is a biomedical literature retrieval system, more than 35 million biomedical articles are included. Therefore, life science researchers cannot keep up with all journals relevant to their areas of interest and select useful information from the latest research. In order to manage biomedical knowledge, curated databases such as UniProt [3], DrugBank [4], CTD [5], and IUPHAR/BPS [6] are constantly being updated. However, updating or developing a database manually can be time-consuming and labor-intensive work, and the speed is often slow, which makes automatic knowledge extraction and mining from biomedical literature highly demanding. Consequently, many pieces of valuable information with complex relationships between entities still remain unstructured and hidden in raw text. 

Recently, AI algorithms have been used to analyze complex forms of medical and life science data to assist human knowledge or to develop protocols for disease prevention and treatment. Moreover, deep learning techniques have been actively applied to various biomedical fields such as drug and personalized medicine development, clinical decision support systems, patient monitoring, and interaction extraction between biomedical entities. For example, protein–protein interaction in biomedical entities are very crucial for understanding various human life phenomena and diseases. Many biochemistry studies go beyond the molecular level of individual genes and focus on the networks and signaling pathways that connect groups or individuals that interact with each other. Similarly, interest in the integration and curation of relationships between biological and drug/chemical entities from text is increasing. 

One of valuable information of drugs and chemical compounds is how they interact with certain biomedical entities, in particular genes and proteins. As mentioned in the study [1], metabolic relations are related to construction/curation of metabolic pathways and drug metabolism such as drug–drug interaction and adverse reactions. Inhibitor/activator associations are related to drug design and system biology approaches. Antagonist and agonist interactions helps in drug design, drug discovery, and understanding mechanism of actions. Drug–drug interaction (DDI) can be defined as a change in the effects of one drug by the presence of another drug. Since such information prevents dangers or side-effects caused by drugs, it is also important to extract useful knowledge from pharmaceutical papers.

**Figure 1:**
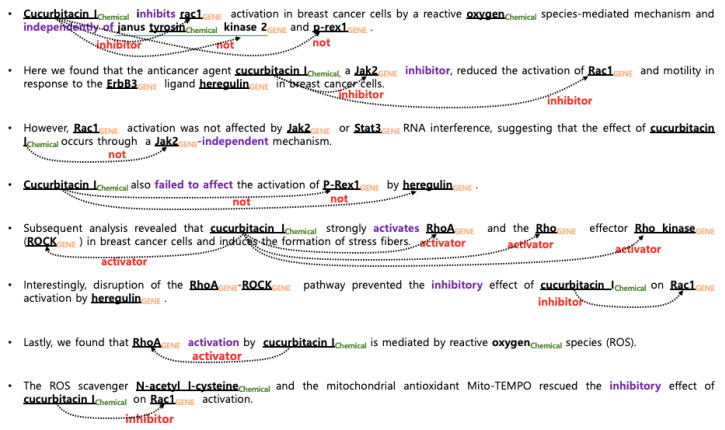
Examples of ChemProt interactions.

**Figure 2:**
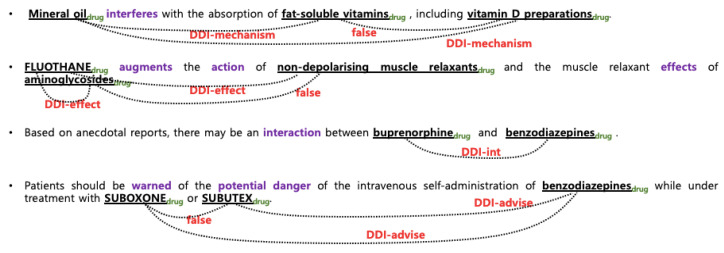
Examples of SemEval13 DDI interactions.

Compared to other fields, texts of biomedical publications are more easily accessible due to the publicly available database MEDLINE [7] and the search system PubMed [2] However, the complexity and ambiguity in biomedical text are much greater than those of general text. One of characteristics of biomedical text is that multiple biomedical entities appear within a single sentence and one entity may be interacted with multiple entities. In particular, it is very difficult to infer which pairs contain actual relations because all entities in a single sentence share the same context, as shown in Figure 1 and Figure 2. In this work, the relation extraction is simplified as classification task, where the problem is to classify which interaction exists between the given pre-recognized entities at sentence level. 

The main objectives of this study are as follows: (1) we apply transfer transformer learning models, which have made impressive performances and progresses in recent years across a wider range of NLP tasks, to the detection of drug-related interactions in biomedical text, and aim to demonstrate which models are effective in biomedical relation extraction. The transformers generate abstract contextual representations of tokens very well by incorporating inter-relations of all tokens in a sequence with the concept of self-attention. As baseline models, three different dominant types of transformers: encoder-only model such as Google’s BERT (Bidirectional Encoder Representations from Transformers) [8], decoder-only model such as OpenAI’s GPT-3 (Generative Pre-trained Transformer) [9], and encoder–decoder structure of Google’s T5 (Text-To-Text Transfer Transformer) [10] are chosen to establish a performance benchmark for our proposed methods. All experiments are conducted using ChemProt corpus [11] and DDI corpus [12] which are a collection of text documents that contains information about chemical/drug–protein/gene interactions and drug–drug interactions, respectively.

(2) The second objective of this study is to investigate the effects of syntactic structure of sentences on biomedical relation extraction by incorporating dependencies between words to enhance self-attention mechanism. According to previous studies, syntactic clues such as grammatical dependencies of a sentence help relation extraction. Some studies [13] have demonstrated that removing tokens outside the subtree rooted at the lowest common ancestor of the two entities or SDP (shortest dependency path) word sequence between two entities from the parse tree can improve relation extraction performance by eliminating irrelevant information from the sentence. However, this simplified representation by considering only the SDP word sequence may fail to capture contextual information, such as the presence of negation, which could be crucial for relation extraction [14].

In this work, we propose BERT_GAT_, a newly developed structure-enhanced encoding model that combines the graph-attention network (GAT) [15] with BERT. We investigate its effectiveness on relation extraction by taking into account not only word token information but also grammatical relevance between words within the attention scheme. To incorporate syntactic information, each dependency tree structure is converted into corresponding adjacency matrix. The GAT model uses an attention mechanism to calculate the importance of words within the input graph. This can allow for the extraction of more relevant information.

(3) Finally, we tailor T5, the encode–decoder transformer which has demonstrated high performances in text generation task, to efficiently handle discriminative, non-autoregressive tasks such as our relation classification problem. Since T5 transformer is designed for text-to-text tasks such as text generation and machine translation, the decoder generates output tokens autoregressively based on previous tokens. This can be less efficient for classification tasks where a single label or output is required. Consequently, decoder’s role is not much in classification tasks. We suggest T5_slim_dec_, which determines the interaction category by removing the self-attention block of T5’s decoder input.

The rest of the paper is organized as follows. In Section 2, related works in the field of biomedical relation extraction is presented. Section 3 briefly describes the dataset and provides necessary background information about transformers to help readers better understand the rest of the paper. Section 4 introduces the baseline models and proposed approaches in detail. Data statistics, results, and analysis are discussed in Section 5, along with comparisons with state of the art approaches and limitations. Finally, conclusions and outlooks are reported in Section 6.

## 2. Related Works

In the DDI (drug–drug interaction) extraction task [12], traditional deep-learning systems, such as convolutional neural networks (CNNs) [16] and recurrent neural networks (RNNs) [17] have shown better performances than feature-based approaches. Recently, the transformer-based models including BERT [8], RoBERTa [18], MASS [19], BART [20], MT-DNN [21], GPT-3 [9], and T5 [10] have demonstrated remarkable improvement in performance across various NLP (Natural Language Processing) tasks by obtaining contextualized token representation through a self-supervised learning on a large-scale raw text such as masked language model. The transformer model is originated from the “Attention Is All You Need” paper [22] researched by Google Brain and Google Research. They also attempted the transfer learning which the weights pretrained on a large-scale text dataset for a specific task such as masked language modeling, next sentence prediction or next token prediction were applied to downstream task by fine-tuning the pretrained models on the downstream task. As a result, pretrained language models tend to perform better than learning new knowledge from scratch with no prior knowledge because they utilize previously learned results.

The pretraining on large-scale raw texts has also significantly improved performance in biomedical domain. BERT based on encoder structure and its variants such as SCIBERT [23], BioBERT [24], and PubMedBERT [25] have been successfully applied in biomedical field. Since previous methods consider only the context around entities in the text, some research has encoded various knowledge besides input tokens, resulting in more informative input representations for downstream tasks [26,27].

Asada et al. [26] explored the impact of incorporating drug-related heterogeneous information on DDI extraction, and achieved an F-score of 85.40. They reported it as state-of-the-art performance. They constructed a HKG (heterogeneous knowledge graph) embedding vectors of drugs by performing a link prediction task which predicts an entity, t, that forms triple (*h*, *r*, *t*) for a given entity, h and relation pair, r on the PharmaHKG dataset. The dataset contains graph information: six nodes (entities), i.e., drug, protein, pathway, category, and ATC (Anatomical Therapeutic Chemical) code, molecular structure from different databases/thesauruses and eight edges (relations): category, ATC, pathway, interact, target, enzyme, carrier, and transporter. The input sentence S was tokenized into sub-word tokens by the BERT tokenizer and extended by adding KG vectors of two drugs. Thus, the input sentence is represented with {[CLS], w_1_, … w_m1_, …, w_m2_, …; [SEP], [KG_m1_] [KG_m2_]}, where w_i_ corresponds to subword and m_1_, to drug_1_ and m_2_, to drug_2_, and [KG_m1_] and [KG_m2_] represent knowledge embeddings for each drug entity.

Similarly, Zhu et al. [28] utilized drug descriptions from Wikipedia and DrugBank to enhance the BERT model with the semantic information of drug entities. They used three kinds of entity-aware attentions to get sentence representation with entity information, mutual drug entity information, and drug entity information. The mutual information vector of two drug entities was obtained by subtracting the BioBERT embeddings of two drugs. For drug description information, all drug description documents were fed into Doc2Vec model and obtained its vector representations for each drug entity appearing in the 2013 DDI corpus. The vectors for entity information were fed into attention layers and retrieve sentence representation vectors integrating entity’s multiple information. They reported 80.9 (micro F1-score) on DDI corpus.

LinkBERT [29] used hyperlinks to create better context for learning general-purpose LMs (language model). The hyperlink can offer new, multi-hop knowledge, which is not available in the single article alone. It creates inputs by placing linked documents in the same context window. They joined the segments of two different documents on BERT via special tokens to form an input instance: [CLS] X_A_ [SEP] X_B_ [SEP], where X_A_ segment belongs to document A and X_B_ segment belongs to document B. They used the Document Relation Prediction (DPR) objective for pretraining, which classifies the relation of two segments X_B_ to X_A_ as contiguous (X_B_ is direct continuation of X_A_), random, and linked. They achieved a performance of 83.35 (micro F1-score) on DDI classification task. SciFive [30] and T5-MTFT [31] pretrained on biomedical text using T5 architecture also showed good performance in relation extraction. In particular, SciFive was pretrained on PubMed abstracts and outperformed other encoder-only models.

## 3. Preliminaries

### 3.1. Data Sets and Target Relations

The evaluation of transformers is conducted on two datasets, namely ChemProt [11] and DDI [12] which are used for RE (relation extraction) between drug-related entities. This paper is not intended to validate different RE methods across various datasets, but rather than focuses on extraction of drug-related interactions and perform a more in-depth evaluation.

In ChemProt track corpus in BioCreative VI, interactions are annotated to explore recognition of chemical–protein relations from abstracts, as shown in Table 1. The corpus contains directed relations from chemical/drug to gene/protein, indicating how the chemical/drug interacts with the gene/protein. Chemical–protein relations, referred to as ‘CPR’, are categorized into 10 semantically related classes that share some underlying biological characteristics. For instance, the interactions such as “activator”, “indirect upregulator” and “upregulator”, which result in an increase in the activity or expression of a target gene or protein, belong to CPR:3 group. The interactions such as “downregulator”, “indirect downregulator”, and “inhibitor” interactions which all decrease the activity or expression of a target gene or protein, belong to CPR:4. For this task, chemical and protein/gene entity mentions were manually annotated. In the track, only relations belonging to the following five classes were considered for evaluation purposes: CPR:3, CPR:4, CPR:5, CPR:6, and CPR:9.

In the DDIExtraction 2013 shared task, five types of interactions are annotated, as shown in Table 2. The false pairs, which are drug pairs that do not interact, were excluded in the evaluation to simplify the evaluation and enable better comparability between systems in the shared task. Table 3 and Table 4 display the number of instances for each class. Figure 1 and Figure 2 illustrate examples of interactions in ChemProt and DDI, respectively. For example, the first sentence in Figure 2 states that ‘mineral oil’ and ‘fat-soluble vitamins’ have a DDI-mechanism relationship, while there is no interaction (false) between ‘fat-soluble vitamin’ and ‘vitamin d preparations’. The interaction between ‘mineral oil’ and ‘vitamin d preparation’ is a DDI-mechanism. Since three interactions appear in one sentence, when creating instances, separators such as ** (## and ** for ChemProt) are added before and after the target entities to indicate the desired interaction pair.

### 3.2. Transformer and Attention

Before explaining our transformer approaches, we will first introduce the concept of the transformer model and attention. The transformer was designed for sequence-to-sequence tasks. It uses stacked self-attentions to encode contextual information of input sequence. Attention is a mechanism which enables a model to focus on relevant parts of the input sequence to enhance the meaning of the word of interest [32]. The inputs to the transformer model are word embedding vectors. The model weighs these vectors according to their neighboring context within the sentence. For example, in the sentence, “He swam across the river to the other bank”, the word, ‘bank’ has a contextualized vector which is closer to the meaning of ‘sloping raised land’ rather than ‘a financial institution’ by focusing on the words “swam” and “river”. 

The attention provides contextualized representation for each word and captures relatedness between other words occurred in the sequence. BERT processes input tokens through transformer encoder blocks and returns a hidden state vector for each token. These hidden state vectors encapsulate information about each input token and the context of the entire sequence.

The attention score, as represented by Equation (1), is computed after creating a query($Q_{i})$, key($K_{i})$, and value($V_{i})$ embedding vector for each token in a sentence. The calculation involves three parts: (1) computing the attention score between query and key using a dot-product similarity function, (2) normalizing the attention score using softmax, and (3) weighting the original word vectors according to surrounding context using the normalized attention weights.

(1) $$Q_{i}=QW_{i}^{Q},K_{i}=KW_{i}^{K},V_{i}=VW_{i}^{V}\;head_{i}=Attention\left(Q_{i},K_{i},V_{i}\right)=softmax\left(\frac{Q_{i}K_{i}^{T}}{\sqrt{d_{k}}}\right)V_{i}\;softmax\left(s_{i}\right)=\frac{e^{s_{j}}}{\sum_{j=1}^{n}e^{s_{j}}}\;Multi_{head\left(Q,K,V\right)}=Concat\left(head_{1},head_{2},\ldots ,head_{h}\right)W^{O}$$

In Equation (1), *d_k_* is the dimension of query/key/value and *n* is the sequence length. The matrix multiplication $QK^{T}$ computes the dot product for every possible pair of queries and keys. If two token vectors are close (similar) to each other, their dot product is going to be big. The shape of each matrix is n×n, where each row represents the attention score between a specific token and all other tokens in the sequence. The softmax and multiplication with value matrices represents a weighted mean and $\sqrt{d_{k}}\;is\;a\;scaling\;factor$. With multi-headed self-attention, multiple sets of Q/K/V weight matrices are used to reflect different representation of the input sequence.

**Figure 3:**
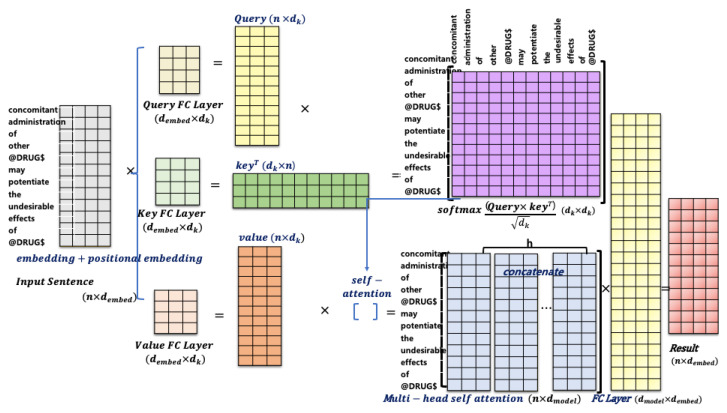
Visualization of multi-head self-attention for an example sentence.

As a result, the attention operation helps focus more on the values associated with keys that have higher similarities and capture important contextual information in the sequence. It produces a contextualized representation of the whole sequence and can be interpreted as connection weights between each word token and all other words in a given sequence. Figure 3 shows how to compute multi-head self-attention for an example sentence: “concomitant administration of other @DRUG$ may potentiate the undesirable effect of @DRUG$.” In the case, “concomitant” might be highly associated with “administration” by the self-attention. The outputs of the attention mechanism are concatenated before being further processed and fed to a FFNN (feed-forward neural network). The transformer encoder takes the input sequence and maps it into a representational space. It generates *d_embed_*-dimensional vector representation for each position of the input, as shown in Figure 3, which is then sent to the decoder.

In addition to word embedding, transformer also employs positional embedding to represent a token’s positional information. This allows for parallel processing with causal masking, which restricts the use of future information during training by masking future tokens that appears after the current position in the input. The positional embedding vector to each input token can be easily computed using sine and cosine functions with Equation (2), where d_model_ represents the dimension of the input embedding vector.

The transformer consists of a stacked encoder and decoder, both of which are built with two sublayers: multi-head self-attention layers as mentioned earlier and fully connected FFN (FeedForward Neural Network) layers. The FFN consists of two linear transformations with the ReLU (Rectified Linear Unit) activation as shown in Equation (3). To prevent the model from losing important features of input data during training, residual connections, as shown in Equation (4), are employed around each of the sub-layers, followed by layer normalization:

(2) $$PE_{\left(pos,2i\right)}=sin(\frac{pos}{10000^{\frac{2i}{d_{model}}}}),{PE}_{\left(pos,2i+1\right)}=cos(\frac{pos}{10000^{\frac{2i}{d_{model}}}})$$

(3) $$FFN\left(x\right)=\max\left(0,xW1+b1\right)W2+b2$$

LayerNorm(x + Sublayer(x))(4)

Besides the two sub-layers, the decoder has an additional sublayer called multi-head cross attentions, which considers the relationship between the output of the encoder and the input of the decoder. The output of the encoder is transformed into a set of K and V vectors and utilized in the cross-attention. The cross attention adopts Q matrix from the self-attention layer of decoder and K and V matrix from the encoder, respectively. Unlike its operation in the encoder, the self-attention layer in the decoder is modified to prevent positions from attending to subsequent positions by masking. This masking ensures that the predictions for position *i* can depend only on the known outputs at positions less than *i*.

In practice, the encoder maps an input sequence to a sequence of continuous contextual representation. Given the input representation, the decoder auto-regressively generates an output sequence, one element at a time, using the previously generated elements as additional input when generating the next.

## 4. Methods

In this section, we first describe three transformers used as baseline models and introduce proposed models, BERT_GAT_ and T5_slim_dec_ for relation extraction.

### 4.1. Baseline Methods

**Figure 4:**
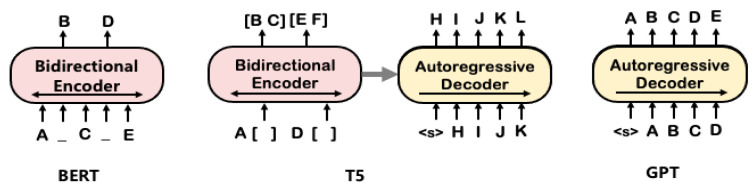
Pretraining methods of transformers.

As baseline models for our research on interaction extraction, we employed three types of transformer: BERT (encoder-only) [8], GPT3 (decoder-only) [9], and T5 (encoder–decoder) [10]. First, BERT is bidirectional transformer which uses only encoder block of the transformer. For a detailed structure and implementation, please refer to the study [22]. BERT is pretrained on two unsupervised tasks: (1) masked language model (MLM), where some of the input tokens are randomly masked and the model is trained to predict the masked tokens and (2) next sentence prediction (NSP), where the model is trained to predict whether one sentence follows another, as shown in Figure 4. It uses WordPiece tokenizer and has a special classification token ‘[CLS]’ in the first token of every sequence which corresponds to the aggregated whole sequence representation.

We initialized the model with SCIBERT [23] for drug-related relationship extraction in order to leverage the domain specific knowledge and then fine-tuned all of the parameters using labeled ChemProt and DDI dataset. SCIBERT has the same architecture as BERT but was pretrained on scientific texts, which consist of 1.14 million papers from the computer science domain (18%) and the broad biomedical domain (82%), sourced from Semantic Scholar [33]. In addition, in-domain WordPiece vocabulary on the scientific corpus was newly constructed. Ultimately, we fed the special ‘[CLS]’ token vector of the final hidden layer into a linear classification layer with softmax output to classify the interaction types.

**Figure 5:**
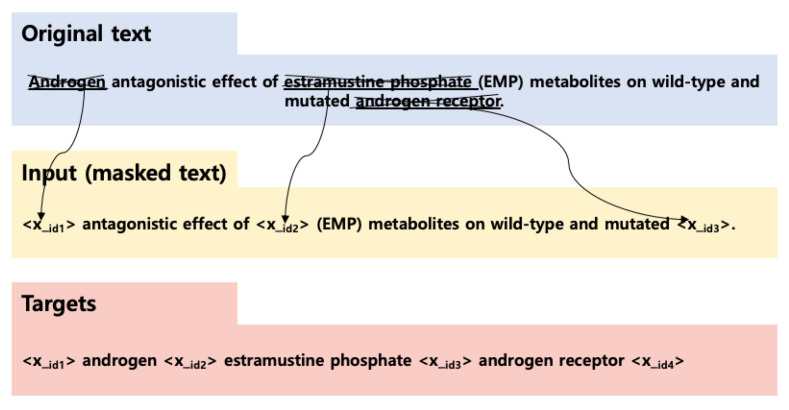
T5’s pretraining scheme.

Secondly, we employed the text-to-text transfer transformer (T5) [10], which is an encoder–decoder model. In the research, the authors experimented with various types of transformers and demonstrated that the encoder–decoder transformer architecture, combined with the denoising (masked language modeling) objective, yielded the best performance for most NLP tasks. T5 was pretrained with self-supervision through a learning objective called span-based language masking, in which a set of consecutive tokens are masked with sentinel tokens and the target sequence is predicted as a concatenation of the real masked spans, as shown in Figure 5. The tokens for pretraining were randomly sampled, and dropped out 15% of tokens in the input sequence. It used SentencePiece tokenizer [34] to encode text.

In general, encoder-only model such as BERT are easily applicable to classification or prediction tasks by using the ‘[CLS]’ token, which provides a summary representation of the entire input sentence. On the contrary, T5 treats every text processing problem into a text-to-text generation problem that takes text as input and produce new text as output. Therefore, our relation classification problem is treated as a generation task for interaction types. Initially, we used the pretrained parameters of the SciFive [30] model and then finetuned it on our specific dataset in relation extraction tasks. The SciFive model was retrained on various text combination, which consisted of the C4 corpus [35], PubMed abstracts, and PMC full-text articles, to optimize the pretrained weights from T5 in the context of biomedical literature. Consistent with the original T5 model [10], SciFive learned to generate a target text sequence for a given text input sequence using a learning objective known as span-based mask language modeling. The output sequence is generated during the decoding phase by applying beam search algorithm. This involves maintaining the top *n* probable output sequences at each timestep and finally generating the output sequence with the highest probability.

Finally, we employed GPT-3 (Generative Pretrained Transformer) [9] which utilizes constrained self-attention where every token can only attend to its left context. As a decoder-only transformer, it was pretrained on a diverse range of web text to predict the next token in an autoregressive manner given the preceding text. It can generate words only conditioned on the left context, so it cannot learn bidirectional interactions.

Previous pretrained models have a limitation in that they need additional large, labeled datasets for a task-specific fine-tuning process to achieve desirable performance. Thus, GPT2 was designed as a general language model for various NLP tasks without the need for extensive fine-tuning. It is capable of performing downstream tasks with little or no fine-tuning, including zero-shot and few-shot learning scenarios, where only a few labeled examples are available for fine-tuning. However, the results were not satisfactory in some tasks. They still need fine-tuning on task-specific labeled data to improve the performance.

In contrast, GPT-3 increased the capacity of transfer language models to 175 billion parameters, thereby allowing the model to utilize its language skills to comprehend tasks with a few examples or natural language instructions. GPT-3 has demonstrated strong performance across a wide range of downstream tasks with a meta-learning technique called ‘in-context learning’, which allows a language model to develop a broad set of skills and policies for tasks and pattern recognition abilities during unsupervised pretraining. This enables the model to rapidly adapt to a desired task during inference time. Its large-scale, autoregressive language model trained on a massive amount of text data has a deep understanding of the rich context of language and enables the model to generate text, which is similar to human writing.

To achieve this, example sequences for various tasks are used as text input to the pretrained model. For instance, sequences for addition can provide a context for performing arithmetic addition, while error correction sequences can demonstrate how to correct spelling mistakes. Given the context, the model can learn how to perform the intended task and utilize the language skills learned during the pretraining phase.

Recently, OpenAI announced ChatGPT (GPT-3.5) and GPT-4, generative AI models based on reinforcement learning from human feedback (RLHF) and ultra-language models, which have shown very impressive results in generating responses. In this paper, we partially evaluated the potential of GPT-3 on relation extraction using GPT-Neo 125 M and GPT-Neo1.3B models [36] which are dense autoregressive transformer-based language models with 125 M and 1.3 billion parameters trained on 8 million web pages.

### 4.2. Self-Attention Using Dependency Graph: BERT_GAT_

In this section, we describe BERT_GAT_ to encode the syntactic structure with graph-attention network (GAT) [15]. It leverages the overall graph structure to learn complex relationships between entities, enabling the classification of various types of relationships. In general, dependency trees provide a rich structure to be exploited in relation extraction. Parse trees can have varying structures depending on the input sentences, which may differ in terms of length, complexity, and syntactic construction. Thus, organizing these trees into a fixed-size batch can be difficult. Unlike linear sequences, where tokens can be easily aligned and padded, the hierarchical structure of parse trees complicates this process. In sequence models, padding is used to create equal-length inputs for efficient batch processing. However, for parse trees, padding is not straightforward, as it involves adding artificial tree nodes that might disrupt the tree’s structure and introduce noise to the model. Due to these difficulties, it is usually hard to parallelize neural models working on parse trees.

On the contrary, models based on the SDP (shortest dependency path) between two entities are computationally more efficient, but they might exclude crucial information by removing tokens outside the path. In addition, some studies stated that not all tokens in the dependency tree are needed to express the relation of the target entity pair. They have utilized SDP [37] or subtree rooted at the lowest common ancestor (LCA) of the two entities [14] to remove irrelevant information. However, SDP can lead to loss of crucial information and easily hurt robustness. For instance, according to the research by Zhang et al. [14], in the sentence “She was diagnosed with cancer last year, and succumbed this June”, the dependency path ‘She←diagnosed→cancer’ is not sufficient to establish that cancer is the cause of death for the subject unless the conjunction dependency to succumbed is also present. In order to incorporate crucial information off the dependency path, they proposed a path-centric pruning strategy to keep nodes that are directly attached to the dependency path.

To address the issue, we here adapt the graph attention network to consider syntactic dependency tree structure by converting each tree into corresponding adjacency matrix. The graph attention [15] is jointly considered in self-attention sublayer to encode the dependency structure between tokens into vector representations. That helps to capture relevant local structures of dependency edge patterns that are informative for classifying relations by considering the relationships between each node and its neighbors, assigning greater weights to more important neighboring nodes. This approach allows for more effective learning of node representations of graph data, ultimately helping to represent node features more accurately.

**Figure 6:**
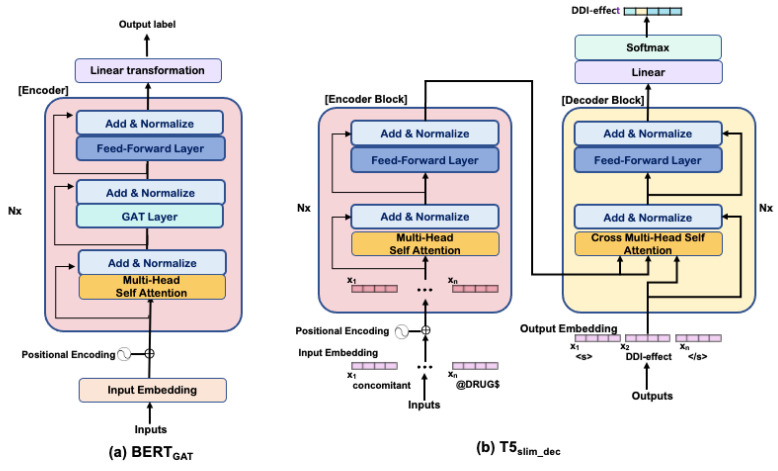
BERT_GAT_ and T5_slim_dec_ architecture.

For this, the Stanford dependency parser [38] is utilized to retrieve universal dependencies for each sentence. A dependency tree is a type of directed graph where nodes correspond to words and edges indicate the syntactic relations between the head and dependent words. In this work, if there is a dependency between node *i* and node *j,* then its opposite direction of dependency, node *j* and node *i* is also included. The dependency types of edge such as ‘subj’ and ‘obj’ are not considered. A self-loop is also considered for each node in the tree. Since BERT takes as subword units generated by tokenizer instead of word-based linguistic tokens of a parse tree, we introduce additional edges to handle unit differences. Figure 6a shows the architecture of BERT_GAT_.

Given a graph with *n* nodes, we can represent the graph with an *n* × *n* adjacency matrix A, where *A_ij_* is 1 if there is a direct edge going from node *i* to node *j*. The encoder consists of two sublayers: multi-head self-attention layer and multi-head self-graph attention layer. The final hidden layer of the encoder is fed into a linear classification layer to predict a relation type, which is followed by a softmax operation. That is, the output layer is one-layer task-specific feed-forward network for relation classification.

The output of the BERT model is a contextualized representation for each word in the given text, which is expressed as the hidden state vector of each word. This output vector contains contextual information about the corresponding word. The input to GAT consists of a set of the hidden state vectors obtained from BERT, $h=\{h_{1},h_{2},\ldots ,h_{V}\}$, which serve as the initial feature vectors for each token in the text.

The GAT layer in Figure 6a produces a new set of node features, $h^{\prime }=\{{h^{\prime }}_{1},{h^{\prime }}_{2},\ldots ,{h^{\prime }}_{V}\}$, as its output and *V* is the number of nodes. The Equations (5) and (6) are used to obtain GAT representation. In this study, we follow the formulation of the Graph Attention Network (GAT) as proposed in the original paper by Veličković et al. (2018) [15]. The GAT model is defined by Equations (5) and (6).

In the beginning, a shared linear transformation, parameterized by weight matrix w is applied to each node to transform the input features into higher-level features. Here, w is a learnable linear projection matrix. Subsequently, a self-attention mechanism a is performed on the nodes and attention coefficients e are computed for every pair of nodes. To calculate the connection importance of node *j* to node *i*, the masked attention coefficient $e_{i,j}$ is computed according to Equation (5) only when *j* is a neighbor of node *i* in the graph. $N_{i}$ represents the set of *i* ‘s one-hop neighbors, including the *i* node itself, as a self-loops are permitted.

While the multi-head self-attention layer in Figure 6a uses a scaled dot product function as a similarity function, the GAT layer uses a one-layer feedforward neural network denoted as a after concatenating the key and query. The scoring function *e* computes a score for every edge (*j,i*), which indicates the importance of the neighbor *j* to the node *i*. It assigns negative value if there is no connection and then the resulting $\alpha_{i,j}$ is normalized with softmax, as shown in Equation (5). It makes the coefficients easily comparable across different nodes. In the equation, the attention mechanism a is a single-layer FFNN, parametrized by a weight vector a and LeakyReLU nonlinearity activation function is applied where T represents transposition and || is the concatenation operation.

(5) $$e_{i,j}=a\left(wh_{i},wh_{j}\right),j\in N_{i}\;a:LeakyReLU(Linear(concat\left(wh_{i},wh_{j}\right))))\;\alpha_{i,j}=softmax\left(e_{i,j}\right)=\frac{exp(e_{i,j})}{\sum_{k\in N_{i}}exp(e_{i,k})}=\frac{exp(LeakyReLU(a^{T}[wh_{i}||wh_{j}]))}{\sum_{k\in N_{i}}exp(LeakyReLU(a^{T}[wh_{i}||wh_{k}]))}$$

The normalized attention coefficients α are used to compute a weighted sum of the corresponding neighbors and to select its most relevant neighbors, as shown in Equation (6). It utilizes the attention mechanism to aggregate neighborhood representations with different weights. That is, each node gathers and summarizes information from its neighboring nodes in the graph. The aggregated information and value is combined and serves as the final output representation for every node. In this way, a node iteratively aggregates the information from its neighbors and updates the representation. To perform multi-head attention, *K* heads are used. Here, σ refers to the ReLU activation function and ${\alpha_{i,j}}^{k}$ means normalized attention coefficients computed by the *k*-th attention mechanism. Finally, we use averaging and activation function and then add a linear classifier to predict for the relation type.

(6) $${h^{\prime }}_{i}=\sigma \left(\sum_{j\in N_{i}}\alpha_{i,j}{Wh}_{j}\right){h^{\prime }}_{i}={\left|\right|}_{k=1}^{K}\sigma \left(\sum_{j\in N_{i}}{\alpha_{i,j}}^{k}W^{k}h_{j}\right)(mutli\_head)\;{h^{\prime }}_{i}=LeakyReLU(\frac{1}{K}\sum_{k=1}^{K}\sum_{j\in N_{i}}\alpha_{i,j}^{k}W^{k}h_{j})$$

**Figure 7:**
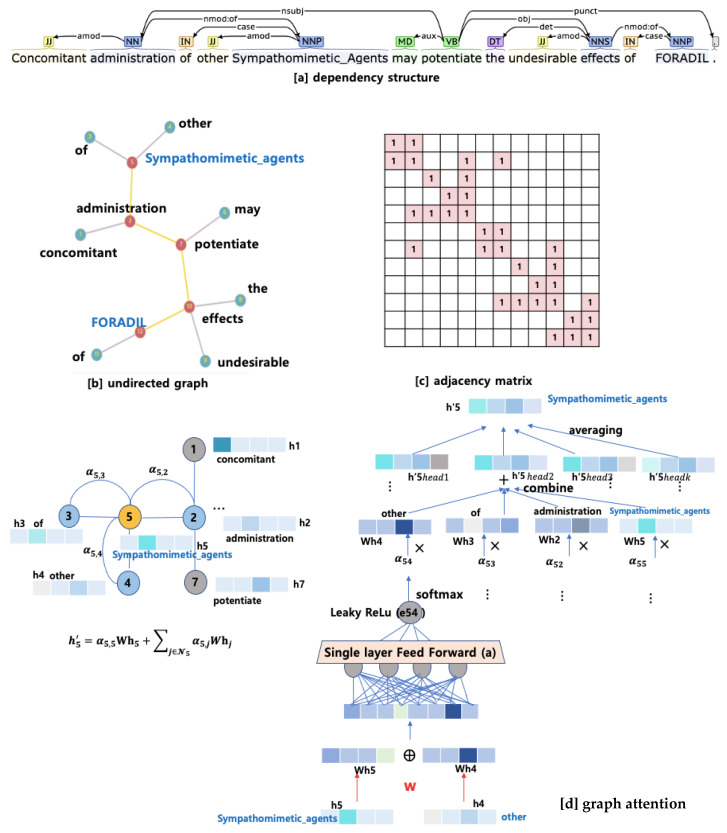
An example of multi-head graph-attention network.

Figure 7 visualizes an example of graph self-attention for an entity node “Sympathomimetic Agents” in the sentence, “Concomitant administration of other Sympathomimetic Agents may potentiate the undesirable effects of FORADL.” The interaction type between the two entities, Sympathomimetic Agents” and “FORDAL” is classified as “DDI-effect”. In the Figure, (a) displays the sentence’s dependency structure, (b) shows the same dependency structure in the form of a graph, (c) presents the adjacency table reflecting the dependency relationships among words, and (d) illustrates the transformation of the vector representation of node 5, “sympathomimetic agents” through graph attention. In addition, this model can incorporate off-connection but useful information by employing a residual connection around each of the two sub-layers, followed by layer normalization. That is, the output of GAT sublayer is LayerNorm(x + GAT_Sublayer(x)), where x is the output of BERT’s self-attention sublayer.

Thus, this model reflects both contextual relatedness and syntactic relatedness between tokens. In addition, the GAT model applies attention to the features of each node’s neighbors to combine them and create a new representation of the node. Therefore, by utilizing attention weights that reflect the importance of edge connections, the neighbor information includes not only directly connected nodes but also indirectly connected nodes, effectively capturing local substructures within the graph.

### 4.3. T5 with Non-Autoregressive Decoder: T5_slim_dec_

As mentioned earlier, T5 [10] converts all text-based language tasks into text-to-text format. As a result, our interaction classification problem is transformed into a relation type generation task, where the model generates a corresponding interaction label between the mentioned entities for a given input sentence. For example, the output label, “DDI-effect” is tokenized as ‘<s>’, ‘_ DD’, ‘I’,’ –‘, ‘effect’, ‘</s>’ and “AGONIST” is as ‘<s>’, ‘▁AG’, ‘ON’, ‘IST’, and ‘</s>‘ in T5. These tokens correspond to decoder’s inputs. Similar to the encoder, the decoder input of target sequence is also embedded, and its positional encoding is added to indicate the position of each word. The self-attention layer in the decoder only allows earlier position tokens to attend to the output sequence by masking future position tokens. This means that the decoder generates output tokens auto-regressively, predicting one token at a time based on the previous tokens, as shown in Equation (7), until a special end symbol, ‘</s>’, is reached indicating the decoder has completed its output. For a given input sequence X, the target sequence Y with a length *m* is generated through a chain of conditional probabilities based on the left-to-right sequential dependencies, where $y_{<i}$ denotes the target tokens up to position *i*.

(7) $$P\left(Y|X\right)=\prod_{i=1}^{m}p(y_{i}|y_{<i},X)$$

The model learns to predict the next token in a sentence more accurately, as it uses teacher forcing to feed the decoder with the actual target tokens from the ground truth data instead of with its own generated previous tokens, during the training phase. The output sequence is generated by searching for the most likely sequences of tokens. By incorporating beam search, T5 can produce more coherent, accurate, and contextually appropriate text outputs. However, to perform classification task under the text-to-text framework, the target label is treated as output text, which is typically a single word or short string. Thus, the autoregressive task, typically used for generating sequences of output text, is not required for class inference. In our work, the output of T5 corresponds to single interaction string, which represents a label such as “DDI-effect” or “AGONIST”. The decoder generates output tokens, each of which represents a specific class from a limited set of class labels. As mentioned in Liu et al.’s study [36], the decoder parameters in T5 model are highly under-utilized for the classification task, in contrast to the typical encoder–decoder models where the decoder layers account for more than half of the total parameters. As a result, when there is only one output token, the decoder has limited previously generated tokens as inputs, which reduces the role of the self-attention mechanism. In such cases, most of the information is passed from the encoder to the decoder and is processed in the cross-attention layer.

Thus, we removed the self-attention block in the decoder, as shown in Figure 6b and tailored the T5 model to fit our interaction-type classification task in a non-autoregressive manner. This approach is inspired by the EncT5 model [39], an encoder-only transformer architecture which reuses T5 encoder layers without code changes. However, we still retained the cross-attention layers to take into account the relationships between the input sentence and output interaction category. The cross-attention plays a role in combining two embedding sequences of the same dimension. It transfers information from an input sequence to the decoder layer to generate output token, which represents the interaction label. The decoder processes the representation of the input sequence through the cross-attention mechanism, yielding a new context-sensitive representation. The embedded vector of the interaction label serves as the query, while the output representation of the encoder is used as both the key and value for the inputs in the cross-attention layer.

For this, we add target labels to vocabulary sets to handle these as whole tokens rather than separated tokens. We also opt for more lexically meaningful labels such as ‘ACTIVATOR’, ‘AGONIST’, ‘AGONIST-ACTIVATOR’, and ‘AGONIST-INHIBITOR’ instead of generic labels such as “CRP:1” or “CRP2”. The model will learn the mapped embedding for this token and the learned embedding will then determine how to optimally pool or aggregate information from the encoder. Finally, the decoder’s output is fed into a linear classifier (a fully connected layer), which transforms the high-dimensional context representation into the size of the number of possible labels. The linear classifier generates decoder_output_logits, which represent the raw and unnormalized output values associated with each label in the vocabulary. The decoder_output_logits are passed through softmax function to convert them into a probability distribution over the entire set of possible labels. The label associated with the highest probability is selected as the output text. We will refer to this model as T5_slim_dec_. Figure 6b presents the overall architecture of T5_slim_dec_.

**Figure 8:**
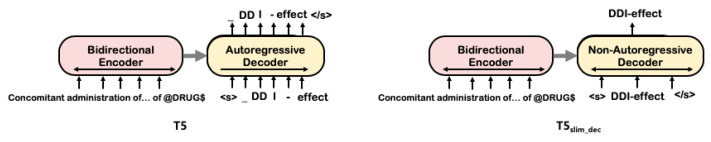
Comparison with T5 and T5_slim_dec_ Models.

Figure 8 visually compares the operational mechanisms of T5 and T5_slim_dec,_ highlighting their differences. As shown in the Figure 8, T5 generates one token at a time based on the input sequence and the previously generated token in the auto-regressive decoding process. For each step of this process, the model calculates decoder_output_logits for all tokens in vocabulary. The token with the highest probability is selected and included in the output sequence and then combines the tokens to form the final readable output text. 

## 5. Results and Discussion

### 5.1. Experimental Setup

In this section, we discuss the results of transformers we suggested in the previous section and how they can be interpreted in comparison to previous studies. All codes were implemented with HuggingFace’s transformers [40] which is a platform that provides APIs and many libraries to access and train state-of-the-art pretrained models. It is available from the HuggingFace hub. We utilized the AdamW optimizer in conjunction with the cross-entropy loss function for training models.

The experimental results were obtained in a GPU-accelerated computing environment using an NVIDIA Tesla V100 32 GB GPU and Google Colab Pro+ with an NVIDIA A100 SXM4 80 GB GPU. To evaluate the model performance, accuracy and F1-score are adopted for evaluation metrics. The accuracy means the proportion of correctly predicted data out of the total data and F1-score is the harmonic mean of precision and recall, designed to balance the two values, as in Equation (8).

(8) $$Accuracy=\frac{TP+TN}{TP+TN+FP+FN}\;Precision=\frac{TP}{TP+FP},\;Recall\left(sensitivity\right)=\frac{TP}{TP+FN}\;F1-score=2\times \frac{Precision\times Recall}{Precision+Recall}$$

### 5.2. Baseline Models

We will begin by presenting the experimental result for the baseline models. In case of encoder–transformer, ‘SCIBERT-uncased’ pretrained model [23] which has the same structure used in BERT [8] were utilized. The model was trained from scratch using the SCIVOCAB, a new WordPiece vocabulary on scientific corpus using the SentencePiece library. Unlike BERT, the model allows maximum sentence length up to 512 tokens. In our relation classification the final vector of the ‘[CLS]’ token was fed into a linear classification layer with softmax outputs to classify interactions. According to the original SCIBERT study [23], the model achieved a micro F1-score of 0.8364 on the ChemProt dataset. However, in our own experiments, we observed a slightly lower performance with 0.8169. In classification tasks for which every case is guaranteed to be assigned to exactly one class, micro-F1 is equivalent to accuracy.

For T5 [10], our tasks were fine-tuned using ‘SciFive-large-Pubmed_PMC’ pretrained model [30]. The model was first initialized with pretrained weights from the base T5 model and then re-trained on C4 [35], PubMed abstracts, and PMC full-text articles. It has 24 decoder/encoder layer and 16 heads. The input length, target length and d_model_ are 512, 16, and 1024, respectively. SciFive [30] used the SentencePiece model [34] for the base vocabulary. Its relation extraction performances on ChemProt and DDI sets were reported as 0.8895 and 0.8367 (micro F1-score), respectively. In our experiment, SciFive pretrained model demonstrated performances of 0.9100 and 0.8808 for the same set. The number of beams was set to 2 during the decoding phase.

In case of GPT-3 model, it is one of the largest generative language models with 175 billion parameters, trained on a massive text data set. It is capable of generating high-quality text on a wide range of tasks. However, GPT-3 is not open-source and is only available through OpenAI’s API. Therefore, for our experiment, we fine-tuned our tasks using EleutherAI’s pretrained models instead. EleutherAI has released several open-source language models called GPT-Neo which perform similarly to GPT-3 but with fewer parameters. Nevertheless, the GPT-NeoX-20B still has 20 billion parameters and requires a large amount of RAM to load the model as well as high-quality computing power to run efficiently. In this experiments, smaller models, such as GPT-Neo1.3b and GPT-Neo125M, were to reduce resource requirements. For future work, the performance of ChatGPT or GPT-4 will be evaluated in the context of biomedical relation extraction to further explore their potential in this domain. Table 5 presents the number of entities in the datasets.

### 5.3. Results of the Proposed Models

Table 6 displays the overall performances (accuracy) of the five attempted methods including BERT_GAT_ and T5_slim_dec_. To simplify parsing and reduce the unnecessary complexity caused by multi-word entity terms in a sentence, entities were masked as entity classes with special @CHEM$ (chemical), @PROT$ (protein), and @DRUG$ (drug) tokens. The term “entity masking” in Table 6 indicates those entity replacements. Experiments were conducted on both original datasets as to which entity mentions are kept and datasets with masked entity names. In general, entity masking is known to be beneficial in the generalization capabilities of relation extraction models by encouraging them to focus on context rather than specific entity mentions. This results in better performance when dealing with new and unseen entities and mitigates the risk of overfitting. In Table 6, it is shown that entity masking in DDI interaction extraction proved to be somewhat effective. On the other hand, in the interaction extraction in ChemProt, using the actual tokens of entities rather than their classes resulted in better performance. One possible reason for this is that the training and evaluation datasets are extracted from the same domain and similar entities are likely to appear more frequently, which can contribute to better performance when not masking entities.

Note that although the ChemProt corpus contains 10 types of relation group classes, only 5 relation types (CPR:3, CPR:4, CPR:5, CPR:6, and CPR:9) were designated to be evaluated in the BioCreative task. In this experiment, two evaluations were conducted: one using the group classes of the CPR-format to which interaction types belong and the other using actual relation types instead of the group classes directly. Consequently, recognizing the interaction class group led to a higher F1-score.

In the case of DDI, the ‘4classes’ in Table 6 indicates that the training and testing were conducted on the four classes (advice, effect, mechanism, int) following the 2013 DDIExtraction shared task evaluation. On the other hand, ‘5classes’ refers to the results of training and testing on the five classes, including ‘DDI-false’. In the table, ‘-false’ indicates the accuracy of interaction labels excluding the cases where the gold label is ‘DDI-false’ during evaluation. In practice, because there were many instances of DDI-false and they were relatively easier to predict, the model achieved a higher F1-score on the 5classes evaluation.

Even though, BERT_GAT_ showed some improvement compared to BERT using entity classes, the performance was still not satisfactory. One reason, the parser is more likely to encounter parsing errors when faced with the complicated biomedical entities and expression. Although the attention mechanism used in GAT allows the model to consider indirectly connected nodes as well as directly connected nodes and BERT’s context representation was used as input feature vector for each node, which make it robust to parsing errors, this method partially depends on a correct parse tree to extract crucial information from sentences. Thus, the accurate performance gain of this approach can be accessed on the availability of human-annotated parses for both training and inference. Currently, the effect of incorporating dependency tree information into pretrained transformer remains uncertain. The BERT_GAT_ was experimented only on ChemProt datasets due to the parsing problem.

Another reason could be that the multi-head attention model based on tokens implicitly encodes syntax well enough since it allows the model to learn from input sequence in multiple aspects simultaneously, with each head collecting information from a different subset. This multi-head structure enables the model to analyze the input from various perspectives and make more accurate predictions without restriction of external dependency structure. Thus, implicit syntactic knowledge within sentences might be learned well by transformer models based solely on tokens.

As a result, T5_slim_dec_ exhibited the best performances on both the ChemProt and DDI datasets and T5 model fine-tuned with SciFive also demonstrated good performances on the datasets. Specially, T5_slim_dec_ demonstrated noticeable improvements in F1-score, compared to the original T5 model. It showed a 6.36% increase from 0.8223 (F1-score) to 0.8746 on the ChemProt task and a 2.4% increase from 0.89 to 0.9115 on the DDI task. The results indicate that the T5_slim_dec_ model is performing well on the interaction classification task by tailoring the decoder structure.

Table 7 and Table 8 show the F1-scores per interaction type. In addition, macro F1-score, micro F1-score, and weighted F1-score were considered as evaluation metrics as well as standard F1-score. Analyzing these metrics can provide a more comprehensive understanding of the models’ performances in multiclass classification by taking into account different aspects of class distribution and the relative importance of each class. In terms of per-class recognition rate, ‘DDI-int’ had the lowest recognition rate in the DDI dataset while ‘‘DOWNREGULATOR’ had lowest recognition rate in the ChemProt dataset. One possible reason for the low performance, the ‘DDI-int’ relation have relatively fewer instances (5.6%) in the DDI corpus compared to other relations. Similarly, the classes ‘AGONIST-ACTIVATOR’, and ‘AGONIST-INHIBITOR’ and ‘SUBSTRATE__PRODUCT-OF’ appeared infrequently in the training dataset, with only 10, 4, and 14 occurrences, respectively. This limited number of examples in the training data may impact the model’s ability to accurately recognize related interactions.

**Figure 9:**
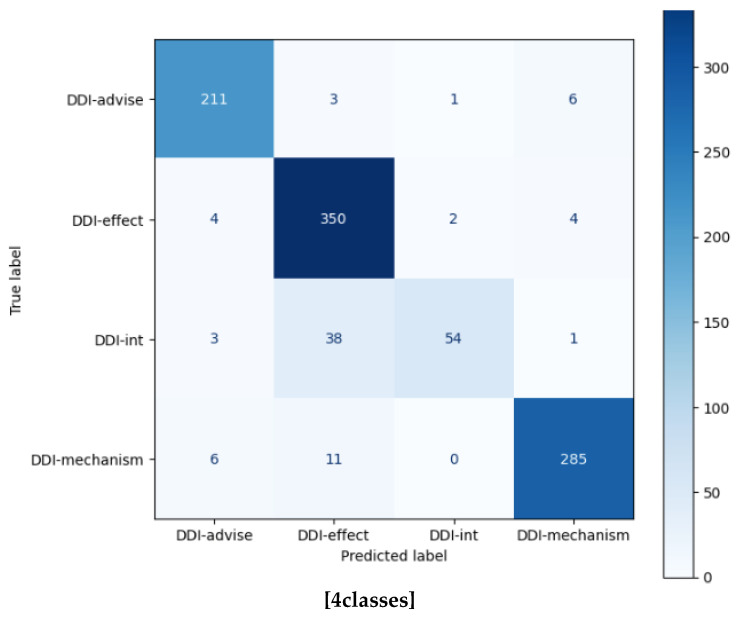

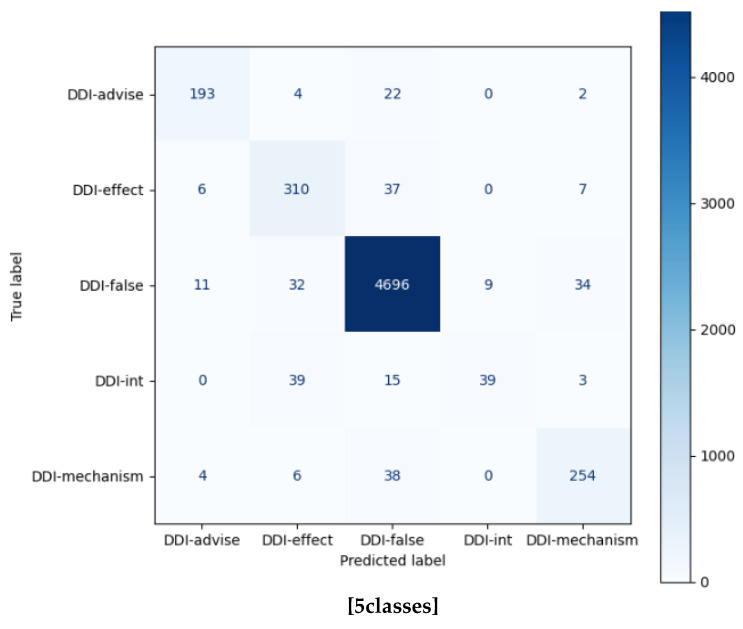
Confusion matrix for T5_slim_dec_ on DDI test dataset.

**Figure 10:**
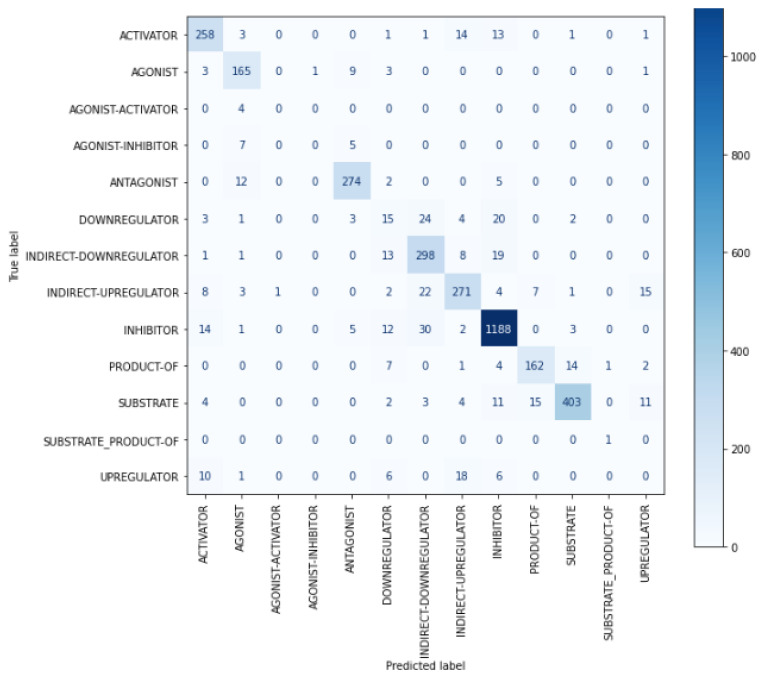
Confusion matrix for T5_slim_dec_ on ChemProt test dataset.

Additionally, Figure 9 shows that ‘DDI-int’ was frequently confused with ‘DDI-effect’ or ‘DDI-false’. The reason may be that this type is assigned when a drug–drug interaction appears in the text without any additional information, which can lead to potential confusion. As shown in Figure 10, ‘DOWNREGULATOR’ interactions in ChemProt dataset were frequently misclassified as different interaction types belonging to the same class group, such as ‘INDIRECT-DOWNREGULATOR’ or ‘INHIBITOR’, as ‘AGONIST-ACTIVATOR’ was often misclassified as ‘AGONIST’ with the same CRP group. Since there might be similarities among them related to their interactions. This makes it difficult for the model to distinguish between them. For example, the ‘DOWNREGULATOR’ represents a chemical that decreases a protein’s activity, while the ‘INHIBITOR’ refers to a chemical that suppresses a specific protein’s function. Both classes have a similarity in that they both decrease or inhibit a protein’s activity.

### 5.4. Comparisons with Other Systems

We also compared T5_slim_dec,_ which showed the best performance, with other previous studies in terms of per-class F1-score per for DDI extraction. As shown in Table 9, T5_slim_dec_ outperformed other two approaches for DDI interaction extraction across all DDI types on the ‘4classes’ evaluation. Additionally, in the ‘5classes’ evaluation, our model performed well compared to others, except for ‘DDI-int’. Since there were limited studies reporting per-class F1-score, few comparisons were presented in Table 9 and Table 10. Zhu et al. [28] constructed three different drug entity-aware attentions to get the sentence representations by using external drug description information, mutual drug entity information, and drug entity information, based on BioBERT. Sun et al. [41] proposed a recurrent hybrid convolutional neural network for DDI extraction and introduced an improved focal loss function to handle class imbalance in the multiclass classification task. 

Table 10 shows the comparison of per class F1-score in the ChemProt dataset. Asada et al. [26] encoded sentence representation vectors by concatenating the drug knowledge graph embedding with word token embedding. The knowledge graph embedding took into account various external information, such as hierarchical categorical information, interacting protein information, related pathway information, textual drug information, and drug molecular structural information. Our T5_slim_dec_ model achieved better classification results for all ChemProt interaction types compared to the current state-of-the-art (SOTA) system [26]. T5_slim_dec_ model with previous systems on DDI and ChemProt relation extraction. Based on the evaluation metric F1-score, our system showed very promising performance in both interaction extraction tasks.

Consequently, T5_slim_dec_ effectively extracted drug-related interactions compared to previous state-of-the-art systems without utilizing external information for entities, simply by tailoring the encoder–decoder transformer architecture to suit the classification task and by not tokenizing the decoder input.

Finally, Table 11 shows an overall performance comparison of our T5_slim_dec_ model with previous systems on DDI and ChemProt relation extraction. The notation ‘CPR’ indicates that the model determines an interaction type by CPR class group, as mentioned earlier. Our experiments showed that SciFive [30], a T5 model trained on large biomedical corpora for domain-specific tasks, performed competitively on both DDI and ChemProt datasets, achieving an accuracy of 0.90 for the 4classes of DDI and 0.91 for the CPR class group of ChemProt. According to our knowledge, SciFive is a state-of-the-art system for drug-related interaction extraction.

As a result, our T5_slim_dec_ model outperformed SciFive with an accuracy of 0.91 for the 4class classification and 0.95 for the 5class classification in the DDI dataset. Additionally, our model achieved an accuracy of 0.94 for the CPR-based class group and 0.87 for 13 interaction types. As shown in the table, encoder-only transformers such as BioBERT, SCIBERT, PubMedBERT, BioM-BERT, and BioLinkBERT exhibited lower performance than encoder–decoder transformer models such as T5 and T5_slim_dec_. Moreover, the PubMedBERT + HKG model, which leverages external knowledge, also showed strong classification accuracy.

### 5.5. Limitations

In this section, we will address several limitations that need to be considered for future improvements. The BERT_GAT_ model encoded dependencies between tokens by converting each tree into a corresponding adjacency matrix. Although the model utilized an attention mechanism to calculate the importance of words within the input graph structure and incorporated BERT’s contextualized representation as embedding feature vectors for input graph nodes, it still requires more sophisticated techniques for incorporating syntactic and semantic information to enhance biomedical relation extraction performance. This is further complicated by errors in the dependency tree which can potentially introduce confusion in relation classification, emphasizing the need for a method that is robust to such issues. Even though the attention mechanism used in GAT allows the model to consider indirectly connected nodes and capture complex relationships in the graph, it is necessary to develop strategies that effectively address these challenges.

In addition, as shown in Figure 10, the T5_slim_dec_ occasionally misclassifies terms with opposite meanings, such as confusing ACTIVATOR with INHIBITOR and AGONIST with ANTAGONIST. This indicates a need for further in-depth research and investigation regarding negation handling to improve the model’s performance in such cases.

Furthermore, due to computing limitations, we were unable to fully validate the performance of GPT-3 in this study, and GPT-Neo1.3b did not outperform the T5 model. Recently, ultra-large language models such as ChatGPT (GPT-3.5) and GPT-4 have demonstrated remarkable performances in text generation. Therefore, further research to explore the potential of ChatGPT or GPT-4 APIs on biomedical interaction extraction is needed.

Finally, the transformer models we proposed were currently designed to perform sentence-level relation extraction, even though transformers can handle multiple sentences simultaneously by using [SEP] to separate them. Thus, they have limitations in handling *n*-ary relation or cross-sentence *n*-ary relation extraction tasks, as there could be more than two entities across multiple sentences.

## 6. Conclusions

In this work, we demonstrated the effectiveness of transfer learning that utilizes transformer models pretrained on a large-scale language dataset and fine-tuned the parameters on relation extraction task dataset.

Although we did not compare the performance of high-capacity parameter models such as GPT-3 or GPT-3.5 (Instruct GPT, ChatGPT) on the relation extraction task, the encoder–decoder transformer T5 consistently demonstrated strong performance in drug-related interaction classification.

We proposed T5_slim_dec_, a modified version of T5 for interaction classification tasks by removing the self-attention layer from the decoder and adding the target labels to the vocabulary. As a result, T5_slim_dec_ can handle the target labels as whole tokens rather than requiring them to be predicted sequentially in an autoregressive manner. The model demonstrates the effectiveness for DDI and ChemProt interaction extraction tasks and achieved improved classification performance compared to state-of-the-art models.

The relation extraction can be a challenging task for transformer models when dealing with complex sentence structures. This difficulty arises from several factors, including long or nested sentences, entities spanning multiple sentences, and domain-specific language structure. To address this difficulty, we incorporated explicit syntactic information to enhance context vector representation of a sentence using structural information of the sentence. We presented BERT_GAT_ to augment the transformer with dependency parsing results. However, that model did not demonstrate a significant performance improvement and additional research is required.

The proposed DDI extraction method can be applied to pharmacovigilance and drug safety surveillance by identifying potential adverse drug interactions. The ChemProt extraction can be utilized in drug discovery and development by facilitating the identification of potential protein targets for new drugs.

## Figures and Tables

**Table 1:** Interaction classes of ChemProt Corpus.

Class Group	ChemProt Relations	Semantic Meaning
**CPR:0**	UNDEFINED	
**CPR:1**	PART-OF	Part-of
**CPR:2**	DIRECT-REGULATOR, INDIRECT-REGULATOR, REGULATOR	Regulator
**CPR:3**	ACTIVATOR, INDIRECT-UPREGULATOR, UPREGULATOR	Upregulator or activator
**CPR:4**	DOWNREGULATOR, INDIRECT-DOWNREGULATOR, INHIBITOR	Downregulator or inhibitor
**CPR:5**	AGONIST, AGONIST-ACTIVATOR, AGONIST-INHIBITOR	Agonist
**CPR:6**	ANTAGONIST	Antagonist
**CPR:7**	MODULATOR, MODULATOR-ACTIVATOR, MODULATOR-INHIBITOR	Modulator
**CPR:8**	COFACTOR	Cofactor
**CPR:9**	SUBSTRATE, SUBSTRATE_PRODUCT-OF, PRODUCT-OF	Substrate or product-of
**CPR:10**	NOT	Not

**Table 2:** Interaction classes of DDI 2013 Corpus.

Relation Class	Semantic Meaning
DDI-Mechanism	a pharmacokinetic interaction mechanism is described in a sentence
DDI-Effect	the effect of an interaction is described in a sentence
DDI-Advice	a recommendation or advice regarding the concomitant use of two drugs is described in an input sentence
DDI-Int	the sentence mentions that interaction occurs and does not provide any detailed information about the interaction
DDI-False	non-interacting entities

**Table 3:** The instances of the ChemProt corpus.

Dataset	CPR:0	CPR:1	CPR:2	CPR:3	CPR:4	CPR:5	CPR:6	CPR:7	CPR:8	CPR:9	CPR:10
train	0	550	1656	784	2278	173	235	29	34	727	242
dev	1	328	780	552	1103	116	199	19	2	457	175
test	2	482	1743	667	1667	198	293	25	25	644	267

**Table 4:** The instances of the DDI extraction 2013 corpus.

Corpus	Advice	Effect	Mechanism	Int	False
train	826	1687	1319	188	15842
test	218	356	302	96	4782

**Table 5:** The number of entities.

Dataset	Entity Type	Number of Entities
**ChemProt**	Protein–Chemical	10,031
**DDI**	Drug–Drug	4920

**Table 6:** Experimental results.

Method	ChemProt Accuracy (Micro F1-Score)			DDI Accuracy (Micro F1-Score)			
	Entity Masking	Actual Relation Type	Class Group (CPR)	Entity Masking	4Classes	5Classes -False	
**SCIBERT**		0.8169	0.8844				
**SCIBERT**	O	0.7852	0.8764	O	0.8703	0.9292	
**BERT_GAT_**	O	0.8089	0.8812				
**GPT-Neo125M**		0.7647	0.8483				
**GPT-Neo1.3b**		0.8204	0.9010		0.8950	0.9261	0.6711
**GPT-Neo1.3b**	O	0.8282	0.9013	O	0.8978	0.9314	0.7263
**T5_sciFive_**		0.8408	0.9100		0.8808	0.9413	0.7268
**T5_sciFive_**	O	0.8223	0.9022	O	0.9031	0.9412	0.7324
**T5_slim_dec_**		**0.8746**	**0.9429**	**O**	**0.9193**	**0.9533**	**0.7998**

**Table 7:** F1-score per DDI type.

Relation Type	4Classes				5Classes			
	Precision	Recall	F1-Score	Support	Precision	Recall	F1-Score	Support
**DDI-advise**	0.9420	0.9548	0.9483	221	0.9019	0.8733	0.8874	221
**DDI-effect**	0.8706	0.9722	0.9186	360	0.7928	0.8611	0.8256	360
**DDI-false**					0.9767	0.9820	0.9794	4782
**DDI-int**	0.9474	0.5625	**0.7059**	96	0.8125	0.4062	**0.5417**	360
**DDI-mechanism**	0.9628	0.9437	0.9532	302	0.8467	0.8411	0.8439	302
**Accuracy**			0.9193	979			0.9533	5761
**Macro avg.**	0.9307	0.8583	0.8815	979	0.8661	0.7927	0.8156	5761
**Weighted avg.**	0.9227	0.9193	0.9151	979	0.9528	0.9533	0.9518	5761
**Micro avg.**	0.9193	0.9193	0.9193	979	0.9533	0.9533	0.9533	5761

**Table 8:** F1-score per ChemProt interaction.

Relation Type	Precision	Recall	F1-Score	Support
**ACTIVATOR**	0.8571	0.8836	0.8702	292
**AGONIST**	0.8333	0.9066	0.8684	182
**AGONIST-ACTIVATOR**	0	0	**0**	**4**
**AGONIST-INHIBITOR**	0	0	**0**	**12**
**ANTAGONIST**	0.9257	0.9352	0.9304	293
**DOWNREGULATOR**	0.2381	0.2083	**0.2222**	72
**INDIRECT-DOWNREGULATOR**	0.7884	0.8765	0.8301	340
**INDIRECT-UPREGULATOR**	0.8416	0.8114	0.8262	334
**INHIBITOR**	0.9354	0.9466	0.941	1255
**PRODUCT-OF**	0.8804	0.8482	0.864	191
**SUBSTRATE**	0.9505	0.8896	0.919	453
**SUBSTRATE_PRODUCT-OF**	0.5	1	**0.6667**	**1**
**UPREGULATOR**	0	0	**0**	**41**
**Accuracy**			0.8746	3470
**Macro avg.**	0.5961	0.6389	0.6106	3470
**Weighted avg.**	0.8682	0.8746	0.8709	3470
**Micro avg.**	0.8746	0.8746	0.8746	3470

**Table 9:** Comparisons of per-class F1-scores with other methods (DDI dataset).

Interaction Type	T5_slim_dec_ (4Classes)	T5_slim_dec_ (5Classes)	Zhu et al. [28]	Sun et al. [41]
**DDI-advise**	0.9483	0.8874	0.860	0.805
**DDI-effect**	0.9186	0.8256	0.801	0.734
**DDI-int**	0.7059	0.5417	0.566	0.589
**DDI-mechanism**	0.9532	0.8439	0.846	0.782

**Table 10:** Comparisons of per-class F1-scores with other method (ChemProt dataset).

Interaction Type	T5_slim_dec_ F1-Score	Asada et al. F1-Score [26]
**ACTIVATOR**	0.8702	0.771
**AGONIST**	0.8684	0.790
**AGONIST-ACTIVATOR**	0	0
**AGONIST-INHIBITOR**	0	0
**ANTAGONIST**	0.9304	0.919
**DOWNREGULATOR**	0.2222	?
**INDIRECT-DOWNREGULATOR**	0.8301	0.779
**INDIRECT-UPREGULATOR**	0.8262	0.752
**INHIBITOR**	0.941	0.853
**PRODUCT-OF**	0.864	0.669
**SUBSTRATE**	0.919	0.708
**SUBSTRATE_PRODUCT-OF**	0.6667	0
**UPREGULATOR**	0	?

**Table 11:** Comparisons with previous SOTA systems.

Method	Accuracy (Micro F1-Score) DDI		Accuracy (Micro F1-Score) ChemProt	
		Our Experiment		Our Experiment
**CNN (Liu et al., 2016)** [16]	0.6701			
**BiLSTM (Sahu and Anand, 2018)** [17]	0.6939			
**BioBERT (Lee et al., 2019)** [24]			0.7646	
**SCIBERT (Beltagy et al., 2019)** [23]			0.8364	0.8169
**BioMegatron (Shin et al., 2020)** [42]			0.77	
**KeBioLM (Yuan et al., 2021)** [27]	0.8190		0.775	
**PubMedBERT(Gu et al., 2021)** [25]	0.8236		0.7724	
**SciFive (Phan et al., 2021)** [30]	0.8367	**0.9031** **_4classes_**	**0.8895**	**0.9100** **_CPR_**
**BioM-BERT (Alrowili et al., 2021)** [43]			0.80	
**BioLinkBERT (Yasunaga et al., 2022)** [29]	0.8335		0.7998	
**PubMedBERT+HKG (Asada et al., 2022)** [26]	**0.8540**			
**BioBERT+multi entity-aware attention (Zhu et al.)** [28]	0.8090			
**Our Method (T5_slim_dec_)**	**0.9533** **_5classes_** **0.9115** **_4classes_**		**0.8746** **0.9429** **_CPR_**	

## Data Availability

Not applicable.
